# Perioperative and Short-Term Outcomes of Sinus Replacement and Conservative Repair for Aortic Root in Acute Type A Aortic Dissection: A Prospective Cohort Study

**DOI:** 10.3389/fcvm.2022.880411

**Published:** 2022-05-19

**Authors:** Yi Chang, Xiangyang Qian, Hongwei Guo, Yizhen Wei, Cuntao Yu, Xiaogang Sun, Bo Wei, Qiong Ma, Yi Shi

**Affiliations:** Department of Vascular Surgery, National Center for Cardiovascular Diseases, Cardiovascular Institute, Fuwai Hospital, Chinese Academy of Medical Science and Peking Union Medical College, Beijing, China

**Keywords:** aortic dissection, aortic root repair, prospective cohort study, propensity score matching, sinus replacement

## Abstract

**Background:**

To compare outcomes between sinus replacement (SR) and conservative repair (CR) for dissected roots with normal size.

**Methods:**

From October 2018 to April 2021, a prospective cohort study was carried out. Patients were assigned to two groups (SR group and CR group) according to whether they underwent sinus replacement. Propensity score matching was applied to adjust preoperative variables and Kaplan–Meier method was used for survival analysis.

**Results:**

Three hundred and eighty-seven patients were enrolled. In the whole cohort, 18 patients (4.7%) died postoperatively. The operative mortality of SR group was comparable to CR group (3.2% vs. 6.0%, *p* = 0.192 before matching; 3.5% vs. 7.0%, *p* = 0.267 after matching) and the incidence of hemostasis management under restarted cardiopulmonary bypass for root bleeding was lower in SR group (1.6% vs. 7.0%, *p* = 0.002 before matching; 2.1% vs. 8.5%, *p* = 0.03 after matching). The median follow-up duration was 12 months. There were 3 reoperations in the CR group. The estimated cumulative event rate of reoperation was 1.1 % at 12 months and 1.6% at 24 months in CR group, with a trend of a lower rate in the SR group (log-rank *p* = 0.089 before matching, *p* = 0.075 after matching). There was one late death in each group. The estimated cumulative event rate of death was 3.8% at 12 months and 24 months in the SR group, and was 6.6% in the CR group with no significant difference (log-rank *p* = 0.218 before matching, *p* = 0.120 after matching). Aortic regurgitation significantly improved postoperatively and remained stable during follow-up.

**Conclusions:**

Sinus replacement is a simple, safe, and effective technique for repairing severely dissected sinus with a comparable time spent in operation and excellent immediate and short-term results. It had the advantages of eliminating false lumen and avoiding aortic root bleeding.

## Introduction

Surgical treatment of acute type A aortic dissection (ATAAD) is challenging and management of the involved aortic root is a key point. In the guidelines, the aortic root involved in dissection should be replaced when its diameter is larger than 45 mm (1). For those aortic roots with normal size, the conservative repair is preferred, accompanied by an estimated freedom from root reoperation of 82–100% at 5 years and 69–93% at 10 years (2). The reoperation was closely related to pathological features and operation choice (3–5). Among various techniques, sinus replacement (SR) (or other names like single patch technique, patch neointima technique) presented with excellent early and long-term results according to our experience and previous research (6, 7). To compare the outcomes between sinus replacement and other conservative repair techniques, we conducted a prospective cohort study from October 2018 to April 2021. In this study, we analyzed the perioperative and short-term outcomes to demonstrate more reliable evidence about aortic root management in ATAAD.

## Materials and Methods

### Study Cohort

The study was approved by the Ethics Committee of Fuwai Hospital in September 2018 and the approval number was 2018-1094. From October 2018 to April 2021, patients with ATAAD were recruited for this prospective cohort study in our institution. The inclusion criteria were as follows: (1) aortic sinus or root being involved; (2) open surgical repair being performed. All the inclusion criteria must be met simultaneously. The exclusion criteria were as follows: (1) having connective tissue diseases; (2) aortic root diameter being more than 45 mm and requiring aortic root replacement; (3) having three aortic sinuses involved simultaneously. One exclusion criterion was enough to reject the candidate. The patients accepted sinus replacement or other conservative repair techniques at surgeons' discretion and then they were assigned to the SR group and CR group. Based on a two-tailed α = 0.05, power (1 – β) = 0.90, and relative risk of aortic root reoperation = 0.125 (based on data from a previous study [2]and our estimation), a sample size of 180 for each group was originally calculated for comparison between the two groups. Taking into account an anticipated 10% loss to follow-up rate, 200 patients were selected for each group to ensure an adequate final sample size. The flow chart of patient enrollment was shown in Supplementary Figure I. Written informed consent was obtained from all participating patients before the start of the study.

### Definition and Classification

Acute aortic dissection was defined as a dissection operated on no later than 14 days after the onset of symptoms. Aortic regurgitation (AR) grade was defined as: 0 = none or trivial, 1 = mild, 2 = moderate, 3 = moderate to severe, and 4 = severe. The classification proposed by Neri et al. (8) was used for coronary artery involvement. Restarted CPB referred to a reoperation under restarting cardiopulmonary bypass (CPB) or cross-clamp for bleeding or myocardial ischemia in the same surgery. The primary end-point was defined as aortic root reoperation during follow-up. The second end-point was defined as death during follow-up.

### Surgical Procedures

A standard median sternotomy was performed for all patients. The strategy of initial arterial cannulation and cardiopulmonary bypass was decided individually, according to the operation method choice, surgeon's preference, and patient's status.

**Figure 1:**
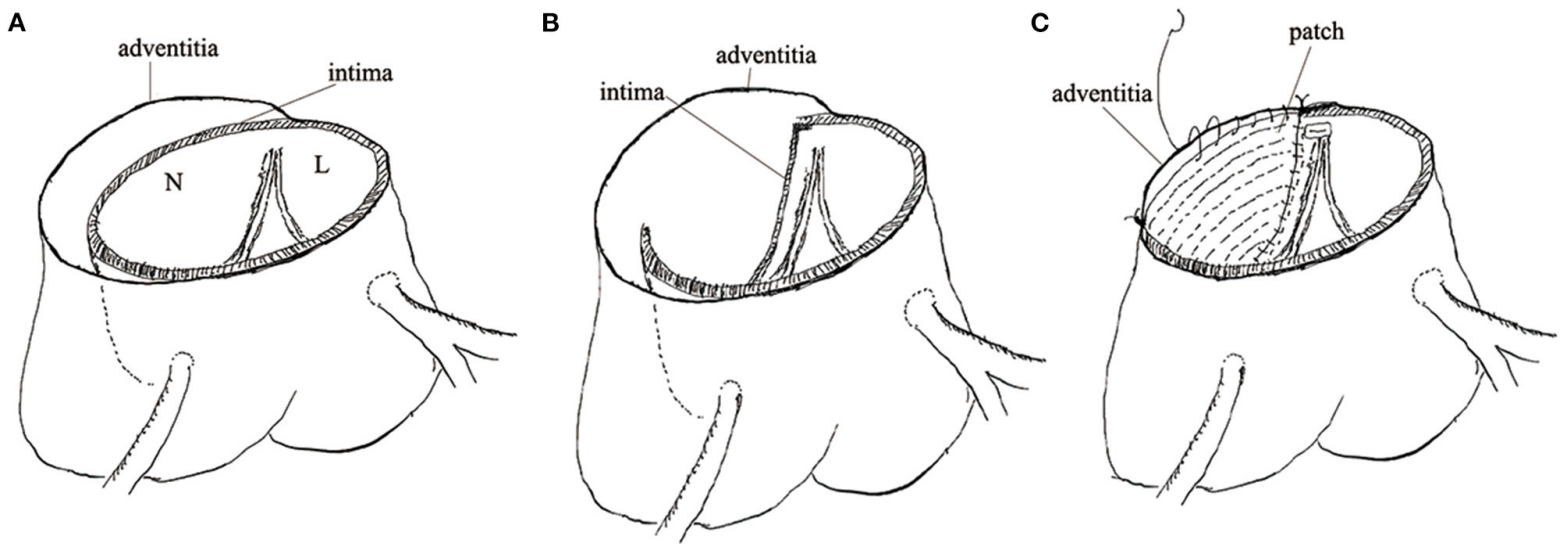
The technical details of sinus replacement. **(A)** Showed deeply dissected non-coronary sinus. **(B)** Showed the intimal flap removed. **(C)** Showed the scallop-shaped patch sewed with remanent intima and adventitia together.

**Figure 2:**
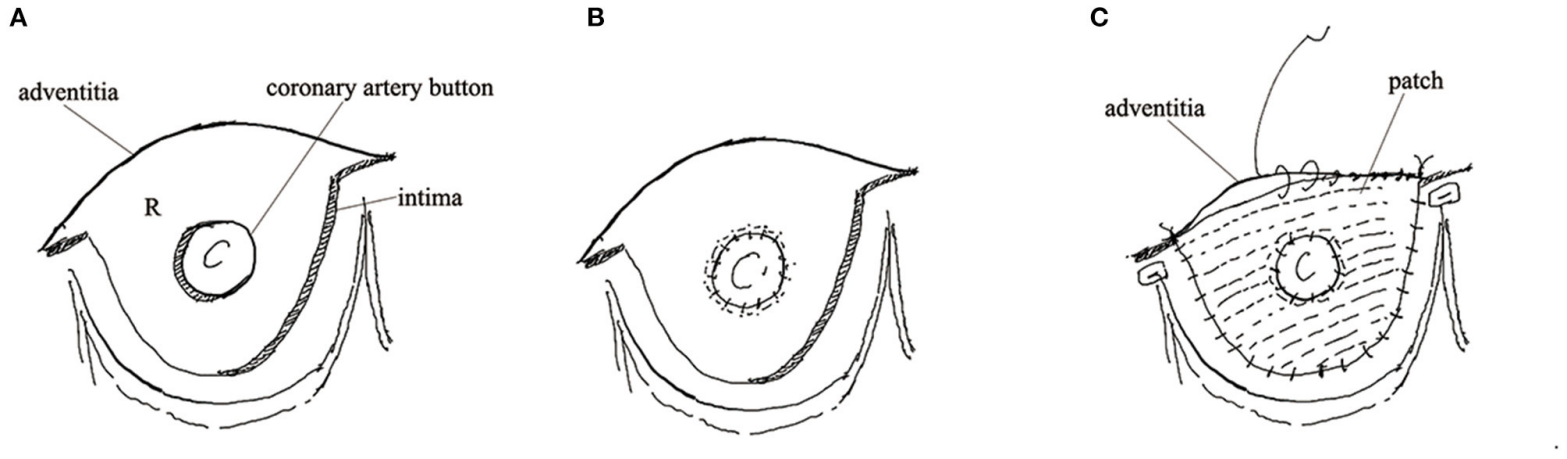
The technical details of sinus replacement. **(A)** Showed the intima of coronary orifice trimmed into button. **(B)** Showed the intimal button attached snugly to the adventitia. **(C)** Showed the patch and the re-implanted button.

Sinus replacement was conducted as follows: ascending aorta was transected 1 cm above sinotubular junction and the intimal flap of the involved sinus was removed maintaining a remanent edge of 5 mm apart from the cusp insertion. Dissecting aortic root from surrounding tissue was troublesome so adventitia was reserved to omit to separate an involved root. A patch deriving from artificial graft was trimmed to a scallop shape similar to native Valsalva sinus. A 5-0 running polypropylene suture was used to sew the patch and remanent intima and adventitia together. Of note, the bottom of the patch should be sewed to the aortic annulus (Figures 1A–C). If we performed sinus replacement in the left or right coronary sinus we would judge the severity of the dissected coronary artery by Neri classification. In type A, the intima of the coronary orifice was trimmed into a button with a 5 mm circumferential cuff. The intimal button was attached snugly to the adventitia by running suture. After sinus replacement was performed as mentioned above, a circular hole was created on the patch for receiving the coronary button. Then the button was re-implanted to the patch using a 5-0 running polypropylene suture (Figures 2A–C). In types B and C, we selected coronary artery bypasses grafting (CABG) for security. The avulsed commissure was attached to adventitia using interrupted mattress suture with the pledge. Then the root stump was prepared for proximal anastomosis. In the CR group, two approaches were used at the surgeon's discretion: neomedia technique and adventitial inversion technique, as the previous study described (9, 10). Neomedia technique was inserting a shaped patch into the false lumen of the root and then intima, the patch, and adventitia were anastomosed with graft. The adventitial inversion technique referred to invert the redundant adventitia overlapping the intima to reinforce anastomosis. These two techniques could reinforce the sutural margin of the aortic root. A total arch replacement was routinely applied in our institution to repair the involved arch. Frozen elephant trunk or endovascular stents were used distally. When the dissection was limited to ascending aorta or proximal arch, isolated ascending aorta replacement or hemi-arch replacement was recommended. Hypothermic circulatory arrest (HCA) and antegrade selective cerebral perfusion were used for arch repair.

### Follow-Up

Data was obtained from each patient's medical chart during regular visits to the outpatient clinic or by telephone contact. The survivors received follow-up using transthoracic echocardiography, CT scan, or both. Survival, reoperation, and AR grade were investigated.

### Statistical Analysis

Data were presented as mean and SD for continuous data conforming to the normal distribution and as number (%) for categorical data. Shapiro–Wilk test was used to evaluate the normality of continuous data. The mean of two continuous normally distributed variables was compared by independent samples Student's *t*-test. Wilcoxon signed-rank test was used to compare non-normal continuous variables. Comparison of categoric variables between groups was analyzed by likelihood ratio Chi-square test or Fisher's exact test.

Propensity score matching was applied to achieve balanced exposure groups at baseline (i.e., minimal confounding). The probability of each patient having an aortic sinus replacement (i.e., the propensity score) was calculated using a logistic regression model. Covariates adjusted and evaluation of propensity score model was demonstrated in Supplementary Table I. Patients were then matched one-to-one using nearest neighbor matching and caliper width of 0.1 of SD of the logit of the propensity score. After propensity score matching, a comparison of continuous data conforming to normal distribution between groups was analyzed by paired *t*-test. Wilcoxon signed-rank test was used to compare non-normal continuous variables. Paired Chi-square test was used to compare multiple categorical variables between the two groups. McNemar test was used to compare binary variables between the two groups. Univariate logistic regression analysis was used to calculate the relative risk (RR) of sinus replacement after matching. Kaplan–Meier method was used for survival analysis and log-rank test was used to compare the difference in cumulative event rate. The competing risk model was constructed by subdistribution hazard function to control the effect of long-term mortality on the primary outcome:long-term risk for reintervention. Statistical significance was denoted by *p*-values < 0.05. The statistical analyses were conducted by SAS (version 9.4, SAS Institute Inc., Cary, NC, USA).

## Results

### Baseline Characteristics and Operative Outcomes

At the end of the study, 387 effective cases were collected, with 187 cases in the SR group and 200 cases in the CR group. In the whole cohort, the average age was 52.3 ±11.5 years, with a male preponderance (68.0%). Coronary artery involvement occurred more frequently in SR group (9.6% vs. 4.0%, *p* = 0.04 for left coronary artery; 38.0% vs. 25.5%, *p* = 0.01 for right coronary artery). The grade of aortic regurgitation differed significantly between the two groups (*p* = 0.003). The patients' baseline characteristics are presented in Table 1. Altogether, 142 pairs of patients were well-matched across groups regarding baseline characteristics, consistent with the right half of Tables 1–3. The absolute standardized mean differences between the two groups regarding baseline characteristics are shown in Supplementary Table II.

**Table 1:** Baseline characteristics.

**Variable**	**Unmatched**			***P***	**Matched**			***P***
	**Overall *n =* 387**	**SR group *n =* 187**	**CR group *n =* 200**		**Overall *n =* 284**	**SR group *n =* 142**	**CR group *n =* 142**	
Age, year (X ±SD)	52.3 ± 11.5	51.6 ±11.0	52.9 ± 11.9	0.27	53.2 ± 11.2	53.2 ± 10.9	53.2 ±11.6	0.98
BMI (X ±SD)	26.6 ±4.3	26.7 ±4.7	26.5 ± 3.9	0.80	26.3 ± 4.4	26.3 ± 4.9	26.4 ± 3.8	
Male (*n*, %)	263(68.0)	130(69.5)	133(66.5)	0.53	194(68.3)	97(68.3)	97(68.3)	1.00
HT (*n*, %)	325(84.0)	150(80.2)	175(87.5)	0.05	239(84.2)	113(79.6)	126(88.7)	0.05
CAD (*n*, %)	60(15.5)	23(12.3)	37(18.5)	0.09	37(13.0)	20(14.1)	17(12.0)	0.72
DM (*n*, %)	12(3.1)	5(2.7)	7(3.5)	0.64	10(3.5)	3(2.1)	7(4.9)	0.34
COPD (*n*, %)	3(0.8)	2(1.1)	1(0.5)	0.61*	3(1.1)	2(1.4)	1(0.7)	1.00
CRI (*n*, %)	9(2.3)	3(1.6)	6(3.0)	0.51*	7(2.5)	3(2.1)	4(2.8)	1.00
Previous heart surgery (*n*, %)	3(0.8)	1(0.5)	2(1.0)	0.22*	3(2.1)	1(0.7)	2(1.4)	0.22
VMS (*n*, %)	3(0.8)	3(1.6)	0(0.0)	0.11*	0(0.0)	0(0.0)	0(0.0)	NA
Initial tear (*n*, %)				0.05				0.98
aAO	289(74.7)	133(71.1)	156(78.0)		220(77.5)	110(77.5)	110(77.5)	
Arch	85(22.0)	50(26.7)	35(17.5)		57(20.1)	28(19.7)	29(20.4)	
DTA	13(3.4)	4(2.1)	9(4.5)		7(2.5)	4(2.8%)	3(2.1)	
Type of CAI (left) (*n*, %)				0.04*				1.00
No	361(93.3)	169(90.4)	192(96.0)		270(95.1)	136(95.8)	134(94.4)	
A	26(6.7)	18(9.6)	8(4.0)		14(4.9)	6(4.2)	8(5.6)	
B	0(0.0)	0(0.0)	0(0.0)		0(0.0)	0(0.0)	0(0.0)	
C	0(0.0)	0(0.0)	0(0.0)		0(0.0)	0(0.0)	0(0.0)	
Type of CAI (right) (*n*, %)				0.01*				0.86
No	265(68.5)	116(62.0)	149(74.5)		202(71.1)	97(68.3)	105(73.9)	
A	98(25.3)	60(32.1)	38(19.0)		65(22.9)	35(24.6)	30(1.1)	
B	17(4.4)	6(3.2)	11(5.5)		11(3.9)	6(4.2)	5(3.5)	
C	7(1.8)	5(2.7)	2(1.0)		6(2.1)	4(2.8)	2(1.4)	
Scr, μmol/L (X ±SD)	96.4 ± 35.8	95.4 ± 37.3	97.4 ± 34.4	0.58	93.4 ± 31.5	89.5 ± 28.8	97.2 ± 33.7	0.03
Lac,mmol/L (media *n*, IQR)	1.47 (1.06–2.17)	1.54 (1.14–2.20)	1.41 (1.03–2.16)	0.28§	1.43 (1.08–2.13)	1.45 (1.14–2.17)	1.38 (1.06–2.03)	0.99§
GPT, IU/L (media *n*, IQR)	22.0 (15.0–36.0)	23.0 (15.0–35.0)	20.0 (14.0–36.0)	0.40§	23.0 (14.0–36.0)	22.0 (14.0–32.0)	25.0 (14.0–37.0)	0.72§
TnI, ng/ml (X ±SD)	0.05 ±0.19	0.05 ±0.11	0.05 ±0.24	0.84	0.05 ±0.16	0.05 ±0.11	0.04 ±0.20	0.58
Root diameter, mm (X ±SD)	37.5 ± 4.3	38.0 ± 4.3	37.1 ± 4.4	0.03	37.7 ± 4.5	38.0 ± 4.3	37.4 ± 4.6	0.09
AR (*n*, %)				0.003*				0.28
No or trivial	176(45.5)	76(40.6)	100(50.0)		124(43.7)	55(38.7)	69(48.6)	
Mild	155(40.1)	75(40.1)	80(40.0)		115(40.5)	57(40.1)	58(40.8)	
Moderate	39(10.1)	28(15.0)	11(5.5)		31(10.9)	23(16.2)	8(5.6)	
Moderate-severe	12(3.1)	8(4.3)	4(2.0)		10(3.5)	7(4.9)	3(2.1)	
severe	3(0.8)	0(0.0)	3(1.5)		2(0.7)	0(0.0)	2(1.4)	

*SR, sinus replacement; CR, conservative repair; HT, hypertension; CAD, coronary artery disease; DM, diabetes mellitus; CRI, chronic renal insufficiency; VMS, visceral malperfusion syndrome; aAO, ascending aorta; DTA, descending thoracic aorta; CAI, coronary artery involvement; Scr, serum creatinine; Lac, lactic acid; IQR, Interquartile Range; GPT, glutamic-pyruvic transaminase; TnI, troponin I; AR, aortic regurgitation.*

*Fisher's exact test was used.

*^§^Wilcoxon signed-rank test was used*.

**Table 2:** Operative characteristics.

**Variable**	**Unmatched**			***P***	**Matched**			***P***
	**Overall *n =* 387**	**SR group *n =* 187**	**CR group *n =* 200**		**Overall *n =* 284**	**SR group *n =* 142**	**CR group *n =* 142**	
SP (*n*, %)	187(48.3)	187(100.0)	0(0.0)	–	142(50.0)	142(100.0)	0(0.0)	–
LCS	17(9.1)	17(9.1)	0(0.0)		13(4.6)	13(9.2)	0(0.0)	
RCS	53(28.3)	53(28.3)	0(0.0)		36(12.7)	36(25.4)	0(0.0)	
NCS	123(65.8)	123(65.8)	0(0.0)		92(32.4)	92(64.8)	0(0.0)	
Commissure reattachment (*n*, %)	142(36.7)	66(35.3)	76(38.0)	0.58	105(37.0)	52(36.6)	53(37.3)	0.90
Arch repair (*n*, %)				0.95*				0.96
None	7(1.8)	4(2.1)	3(1.5)		7(1.8)	4(2.8)	3(2.1)	
HAR	17(4.4)	8(4.3)	9(4.5)		11(3.9)	5(3.5)	6(4.2)	
TAR	363(93.8)	175(93.6)	188(94.0)		266(93.7)	133(93.7)	133(93.7)	
DTA management (*n*, %)				0.08				0.60
None	48(12.4)	20(10.7)	28(14.0)		38(13.4)	16(11.3)	22(15.5)	
FET	308(79.6)	157(84.0)	151(75.5)		224(78.9)	116(81.7)	108(76.1)	
Endovascular stent	31(8.0)	10(5.3)	21(10.5)		22(7.7)	10(7.0)	12(8.5)	
CABG (*n*, %)	64(16.5)	34(18.2)	30(15.0)	0.40	47(16.5)	28(9.7)	19(13.4)	0.16
CPB duration, min (X ±SD)	183.5 ± 71.6	184.7 ± 61.9	182.5 ± 79.7	0.76	185.8 ± 74.0	185.7 ± 62.0	185.8 ± 84.6	0.99
Cross-clamp duration, min (X ±SD)	112.2 ± 41.9	114.7 ± 43.6	109.9 ± 40.3	0.26	113.1 ± 39.7	114.3 ± 39.9	112.0 ± 39.6	0.62
HCA duration, min (X ±SD)	14.0 ± 9.3	14.3 ± 8.8	13.7 ± 9.7	0.53	13.6 ± 9.3	13.9 ± 9.2	13.6 ± 9.4	0.94
Operation duration, hour, (X ±SD)	6.4 ± 1.9	6.3 ± 1.7	6.5 ± 2.0	0.20	6.4 ± 1.9	6.3 ± 1.7	6.4 ± 2.1	0.46

*SR, sinus replacement; CR, conservative repair; LCS, left coronary sinus; RCS, right coronary sinus; NCS, none coronary sinus; HAR, hemi-arch replacement; TAR, total arch replacement; DTA, descending thoracic aorta; FET, frozen elephant trunk; CABG, coronary artery bypass grafting; CPB, cardiopulmonary bypass; HCA, hypothermic circulatory arrest.*

*Fisher's exact test was used.

**Table 3:** Perioperative outcome characteristics.

**Variable**	**Unmatched**			***P***	**Matched**			***P***
	**Overall *n =* 387**	**SR group *n =* 187**	**CR group *n =* 200**		**Overall *n =* 284**	**SR group *n =* 142**	**CR group *n =* 142**	
MV duration, hour (median, IQR)	21.0 (13.0–44.0)	21.0 (13.0–56.0)	21.0 (13.0–41.5)	0.34§	19.0 (13.0–40.0)	20.0 (13.0–43.0)	18.5 (13.0–40.0)	0.59§
ICU stay, day (X ±SD)	5.4 ± 4.7	5.5 ± 5.1	5.3 ± 4.2	0.66	5.0 ± 4.3	5.0 ± 4.8	5.0 ± 3.7	0.97
Operative mortality (*n*, %)	18(4.7)	6(3.2)	12(6.0)	0.19	15(5.3)	5(3.5)	10(7.0)	0.27
Restarted CPB for root bleeding (*n*, %)	17(4.4)	3(1.6)	14(7.0)	0.002	15(5.3)	3(2.1)	12(8.5)	0.03
PMI (*n*, %)	1(0.3)	0(0.0)	1(0.5)	1.00*	1(0.3)	0(0.0%)	1(0.7)	1.00
Reoperation for bleeding (*n*, %)	7(1.8)	2(1.1)	5(2.5)	0.45*	2 (0.7)	0(0.0)	2(1.4)	0.50
IABP (*n*, %)	1(0.3)	0(0.0)	1(0.5)	1.00*	1(0.4)	0(0.0)	1(0.7)	1.00
ECMO (*n*, %)	4(1.0)	0(0.0)	4(2.0)	0.12*	4(1.4)	0(0.0)	4(2.8)	0.13
Stroke (*n*, %)	8(2.1)	1(0.5)	7(3.5)	0.07*	5 (1.8)	0(0.0)	5(3.5)	0.06
CRRT (*n*, %)	23(5.9)	12(6.4)	11(5.5)	0.70	14(4.9)	7(4.9)	7(4.9)	1.00
Paraplegia (*n*, %)	6(1.6)	0(0.0)	6(3.0)	0.03*	3(1.1)	0(0.0)	3(2.1)	0.25

*SR, sinus replacement; CR, conservative repair; MV, mechanical ventilation; IQR, Interquartile Range; ICU, intensive care unit; CPB, CABG, coronary artery bypass grafting; PMI, myocardial infarction; IABP, intra-aortic balloon pump implantation; ECMO, extracorporeal membrane oxygenation; CRRT, continuous renal replacement therapy.*

*^§^Wilcoxon signed rank test was used.*

*Fisher's exact test was used.

In the SR group, 181 patients had one sinus replacement and 6 patients had two sinuses replacements. The noncoronary sinus was most frequently involved and was replaced in 123 patients. In the whole cohort, 64(16.5%) patients underwent CABG for coronary artery involvement or coronary artery disease. All the concomitant operations were distributed similarly between the two groups. The main time variables including CPB duration, cross-clamp duration, HCA duration, and operation duration were comparable with no significant difference. Operative characteristics are listed in Table 2.

The operative mortality of the whole cohort was 4.7% (18 patients), 6 in the SR group and 12 in the CR group. The main presumed causes of death were acidosis and multiple organ failure (8 cases), circulatory failure (5 cases), and stroke (5 cases). The operative mortality of SR group was comparable to CR group (3.2% vs. 6.0%, *p* = 0.192 before matching; 3.5% vs. 7.0%, *p* = 0.267 after matching). Significantly fewer patients needed restarted CPB for root bleeding in SR group (1.6% vs. 7.0%, *p* = 0.002 before matching; 2.1% vs. 8.5%, *p* = 0.03 after matching). The other perioperative outcomes are demonstrated in Table 3. There was no significant association between sinus replacement and operative mortality after matching (RR = 0.48; 95% CI 0.16–1.45). A comparison of operative and perioperative outcomes across the three root management groups is shown in Supplementary Table IV.

### Follow-Up Results

**Figure 3:**
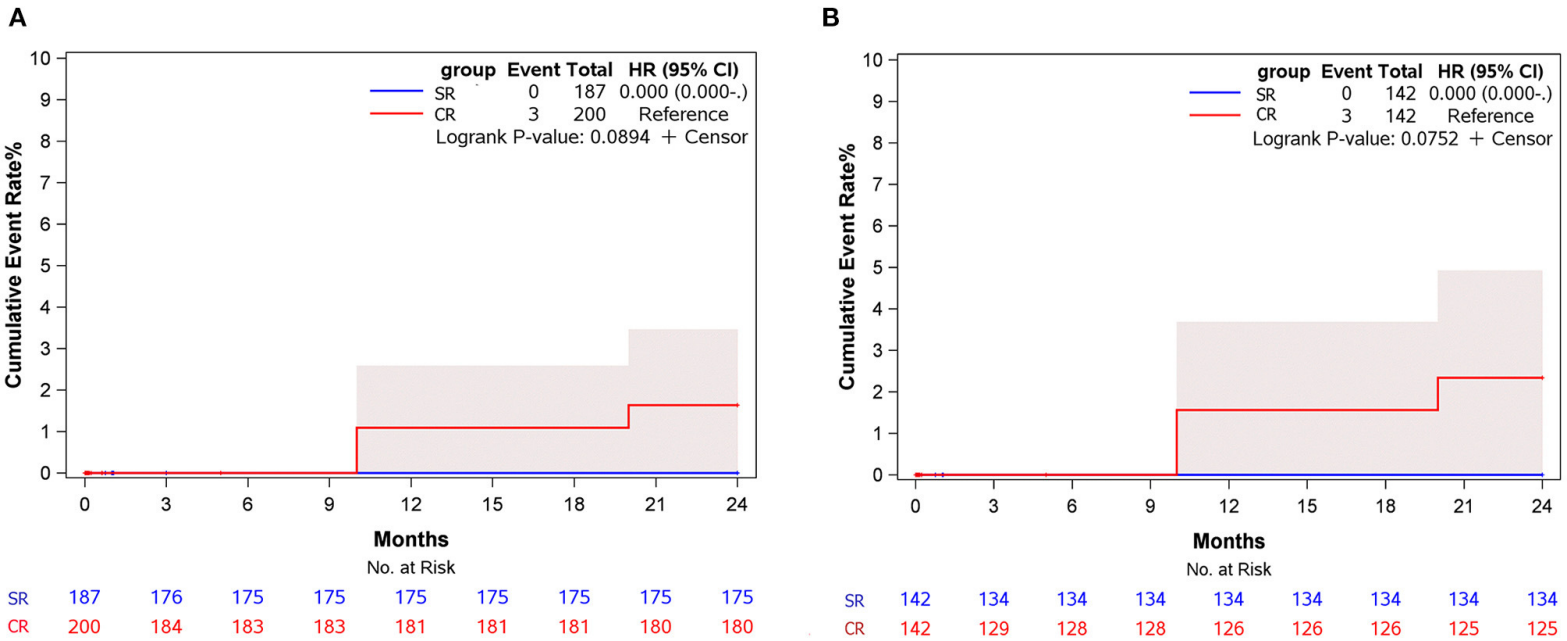
Cumulative event rate of reoperation at 24 months of follow-up for subjects in different surgical groups. **(A)** Before matching. **(B)** After matching.

**Figure 4:**
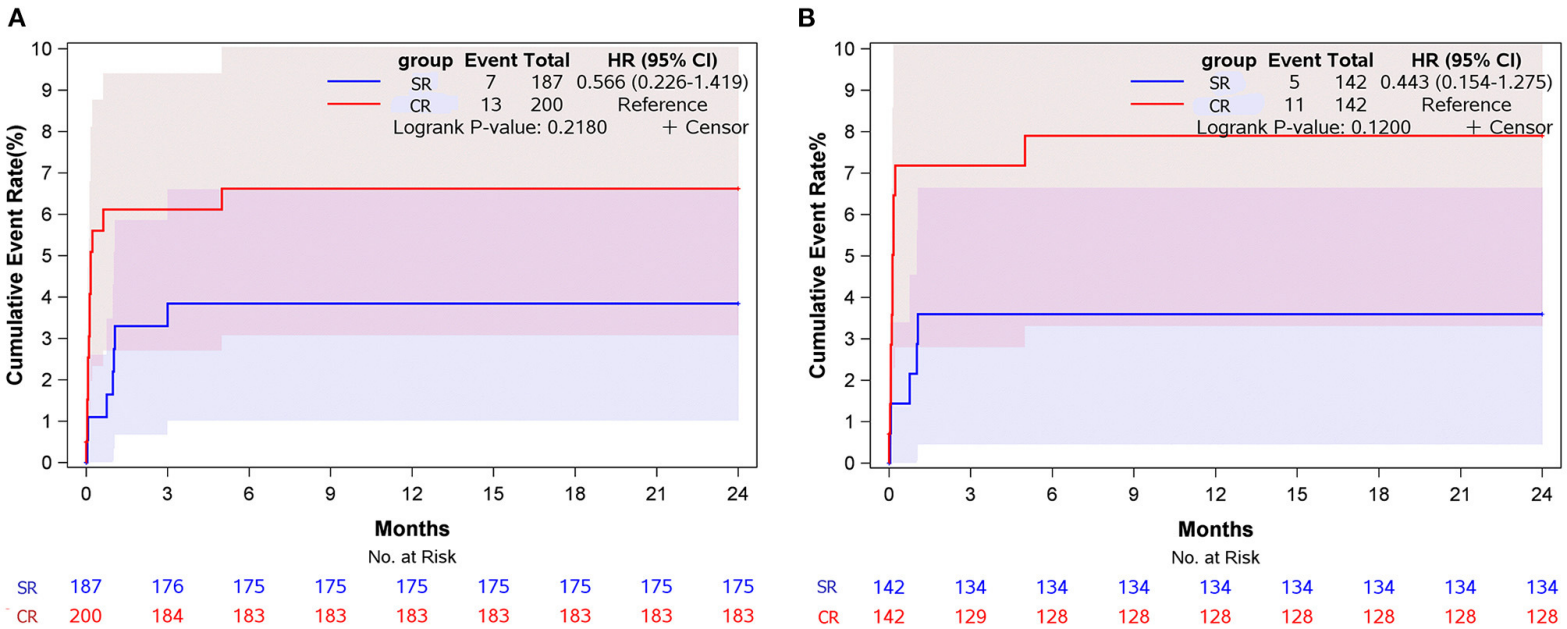
Cumulative event rate of death at 24 months of follow-up for subjects in different surgical groups. **(A)** Before matching. **(B)** After matching.

During a median follow-up of 12 (Interquartile Range [IQR] 9–17) months, five and four patients were lost to follow-up in the SR group and CR group, respectively, and 20 patients were lack of latest echocardiography data. There was one late death in each group (unknown reason) and 3 patients received reoperation due to residual aortic root dissection in the CR group. No patients had aortic regurgitation more than moderate during follow-up in both groups. Postoperative deaths were included in the estimation of long-term mortality. No reoperation occurred in the SR group. The estimated cumulative event rate of reoperation was 1.1 % at 12 months and 1.6% at 24 months in the CR group (Figure 3A). The estimated cumulative event rate of death was 3.8% at 12 months and 24 months in SR group, and was 6.6% at 12 months and 24 months in the CR group (Figure 4A), respectively. No matter before or after matching, there was no significant difference in the long-term cumulative event rate of reoperation in the SR group (log-rank *p* = 0.089 before matching, *p* = 0.075 after matching), and no significant difference in the cumulative event rate of death among the two groups (log-rank *p* = 0.218 before matching, *p* = 0.120 after matching), as shown in Figures 3, 4. In competing risk analysis, there was no significant difference in the cumulative event rate of reoperation after controlling the effect of long-term mortality (Multivariate subdistribution hazard model analysis can be seen in Supplementary Table III and survival curves are shown in Supplementary Figure II).

## Discussion

This study is important because it compares different aortic root repair strategies prospectively. The main findings of our study can be summarized as follows:

1) Regarding pathological features of dissection, sinus replacement was a safe and simple technique with a comparable operative mortality and time spent in operation.
2) Sinus replacement had an advantage in decreasing severe aortic root bleeding that needed to be restarted cardiopulmonary bypass.
3) In short-term follow-up, there was no reoperation in the sinus replacement group.

Even in the third decade of the twenty-first century, treatment of ATAAD is also a challenging task. The management of aortic roots is crucial. For those people with connective tissue disease or a large root (diameter > 45mm), aortic root replacement is appropriate (1).

When the aortic root can be preserved, restoring aortic valve competency and avoiding catastrophic bleeding should be considered. To eliminate residual dissection and avoid proximal catastrophic bleeding due to vulnerable tissue, multiple root repair techniques using prosthetic and biologic materials have been reported (6, 7, 9, 10).

In our center, we carried out sinus replacement for the patients with severely dissected sinus and preservable roots. This technique had remarkable feasibility because tailoring and suturing the patch were simple and easily observable. The thin and flexible vascular graft patch had no influence on root morphology. We could add stitches on the patch without concerning frail adventitia tearing and catastrophic bleeding. Differing from other methods, we just removed intima but reserved adventitia on basis of two considerations: first, the avulsed intima had lost its structural function and could not hold the suture well; second, dissecting adventitia from surrounding tissues was time-consuming and might lead to adventitia rupture and subsequent bleeding.

In this prospective cohort study, the duration of CPB, cross-clamp HCA, and operation in the SR group was no longer than that of the CR group. The time variables were comparable to Urbanski's study (6). It can be believed that sinus replacement was feasible without wasting time.

Overall, compared with previous studies (6, 7, 9, 10), we had a lower operative mortality and a comparable incidence of main complications in this study. The operative mortality was comparable between the two groups before and after matching. We considered that perioperative death was multifactorial in ATAAD treatment. In this study, management of aortic root was not a risk factor for death after adjusting for other lethal comorbidities. Coronary malperfusion was an independent risk factor of mortality (11) and a successful management of the involved coronary artery was necessary for rescuing patients. The management of the involved coronary artery was reported by several studies (8, 12, 13). For the patients whose coronary orifice was dissected circumferentially, we selected reimplantation of the coronary orifice to the patch in the SR group. The intima of the coronary orifice was trimmed into a button without separating adventitia from surrounding tissue. This was a more precise repair of the coronary orifice. In the CR group, intermittent pledgetted stitches around the orifice or CABG were either choice for circumferentially dissected coronary orifice. According to our strategy, CABG was used as a standard approach for main trunk dissection and detachment of orifice. Fortunately, there were no coronary artery-related events and deaths in both groups. Sinus replacement did not increase operative mortality.

It seemed to be expected that a significantly lower incidence of root bleeding requiring restarted CPB occurred in the SR group. We considered that the intima completely detaching from adventitia was not strong enough to hold the suture. If the intima was not removed completely, once anastomotic bleeding occurred, adding stitches on fragile adventitia and intima would lead to catastrophic bleeding. The sinus replacement technique could avoid these troubles to some extent. Because we preserved adventitia and sutured it with patch together, there was also a space between adventitia and patch. Tight stitching was needed to prevent blood from leaking into this space.

Regarding follow-up, we just presented short-term results. Three patients received reoperation of aortic root due to residual dissection in the CR group. In the SR group no reoperation occurred. There were no significant differences in the cumulative event rate of reoperation between the two groups, even after adjusting by competing risk analysis. This might attribute to the short follow-up time and few reoperation events. The excellent mid-and long-term result of a similar technique was found in Irimie et al. report (14). In their study, there was no aortic root reoperation during follow-up with a mean duration of 70 months. As we speculated before, complete removal of dissected intima could eliminate residual false lumen of the aortic root and decrease the risk of reoperation.

Mazzucotelli et al. (15) found that preservation of the aortic valve during surgery for ATAAD may be a valuable choice regardless of the severity of AR. The same conclusion was drawn by Rylski et al. (9) and Ro et al. (16). Indeed, aortic regurgitation secondary to aortic dissection is commonly due to detachment of aortic valve commissure and resuspension of the commissures could typically preserve aortic valve competency. In this study, as many as 36.7% of patients received commissure reattachment. The grade of AR significantly improved postoperatively and remained stable during follow-up no matter what approaches were used to repair the root. In the present study, the follow-up duration was short and the stability of aortic valve function needed long-term observation.

This study has some limitations. First, up to 8 surgeons attended this study and performed these procedures, so the differences in technique might increase the uncertainty of the results. Second, the follow-up duration was short and long-term results needed to be determined in the future.

## Conclusion

Sinus replacement was a safe and simple technique with a comparable operative mortality and time spent in operation. It also had an excellent immediate and short-term results. It had the advantages of eliminating false lumen, preventing severe root bleeding, and might avoid residual aortic root dissection and reoperation.

## Data Availability Statement

The raw data supporting the conclusions of this article will be made available by the authors, without undue reservation.

## Ethics Statement

The studies involving human participants were reviewed and approved by Ethics Committee of Fuwai Hospital. The patients/participants provided their written informed consent to participate in this study.

## Author Contributions

YC: data curation and writing—original draft. XQ: conceptualization, methodology, investigation, and funding acquisition. HG, YW, CY, XS, BW, QM, YS: investigation. All authors contributed to the article and approved the submitted version.

## Funding

This work was supported by Beijing Municipal Science and Technology Commission (No. Z181100001718166).

## Conflict of Interest

The authors declare that the research was conducted in the absence of any commercial or financial relationships that could be construed as a potential conflict of interest.

## Publisher's Note

All claims expressed in this article are solely those of the authors and do not necessarily represent those of their affiliated organizations, or those of the publisher, the editors and the reviewers. Any product that may be evaluated in this article, or claim that may be made by its manufacturer, is not guaranteed or endorsed by the publisher.

## References

[1] Hiratzka LF Bakris GL Beckman JA Bersin RM Carr VF Casey DE. 2010 ACCF/AHA/AATS/ACR/ASA/SCA/SCAI/SIR/STS/SVM guidelines for the diagnosis and management of patients with Thoracic Aortic Disease: a report of the American College of Cardiology Foundation/American Heart Association Task Force on Practice Guidelines, American Association for Thoracic Surgery, American College of Radiology, American Stroke Association, Society of Cardiovascular Anesthesiologists, Society for Cardiovascular Angiography and Interventions, Society of Interventional Radiology, Society of Thoracic Surgeons, and Society for Vascular Medicine. Circulation. (2010) 121:e266–369. 10.1161/CIR.0b013e3181d4739e 20233780

[2] Trimarchi S Eagle KA Nienaber CA. Role of age in acute type A aortic dissection outcome: report from the International Registry of Acute Aortic Dissection (IRAD). J Thorac Cardiovasc Surg. (2010) 140:784e9. 10.1016/j.jtcvs.2009.11.014 20176372

[3] Tan ME Morshuis WJ Dossche KM Kelder JC Waanders FG Schepens MA. Long-term results after 27 years of surgical treatment of acute type a aortic dissection. Ann Thorac Surg. (2005) 80:523–9. 10.1016/j.athoracsur.2005.02.059 16039197

[4] Concistrè G Casali G Santaniello E Montalto A Fiorani B. Dell'Aquila A, Musumeci F. Reoperation after surgical correction of acute type A aortic dissection: risk factor analysis. Ann Thorac Surg. (2012) 93:450–5. 10.1016/j.athoracsur.2011.10.059 22206955

[5] Kirsch M Soustelle C Houël R Hillion ML Loisance D. Risk factor analysis for proximal and distal reoperations after surgery for acute type A aortic dissection. J Thorac Cardiovasc Surg. (2002) 123:318–25. 10.1067/mtc.2002.119702 11828292

[6] Urbanski PP Lenos A Irimie V Bougioukakis P Zacher M Diegeler A. Acute aortic dissection involving the root: operative and long-term outcome after curative proximal repair. Interact CardioVasc Thorac Surg. (2016) 22:620–6. 10.1093/icvts/ivw002 26848190 PMC4892147

[7] Chen LW Wu XJ Li QZ Dai XFA. modified valve-sparing aortic root replacement technique for acute type A aortic dissection: the patch neointima technique. Eur J Cardiothorac Surg. (2012) 42:731–3. 10.1093/ejcts/ezs371 22743079

[8] Neri E Toscano T Papalia U Frati G Massetti M Capannini G. Proximal aortic dissection with coronary malperfusion: presentation, management, and outcome. J Thorac Cardiovasc Surg. (2001) 121:552–60. 10.1067/mtc.2001.112534 11241091

[9] Rylski B Bavaria JE Milewski RK Vallabhajosyula P Moser W Kremens E. Long-term results of neomedia sinus valsalva repair in 489 patients with type A aortic dissection. Ann Thorac Surg. (2014) 98:582–8. 10.1016/j.athoracsur.2014.04.050 24928674

[10] Tanaka K Morioka K Li W Yamada N Takamori A Handa M. Adventitial inversion technique without the aid of biologic glue or Teflon buttress for acute type A aortic dissection. Eur J Cardiothorac Surg. (2005) 28:864–9. 10.1016/j.ejcts.2005.08.029 16275115

[11] Czerny M Siepe M Beyersdorf F Feisst M Gabel M Pilz M et al. Prediction of mortality rate in acute type A dissection: the German Registry for Acute Type A Aortic Dissection score. Eur J Cardiothorac Surg. (2020) 58:700–6. 10.1093/ejcts/ezaa156 32492120

[12] Tang YF Zhang GX Liao ZL Han L Xu ZY. Surgical treatment of coronary malperfusion with acute type A aortic dissection. Chin Med J. (2016) 129:1000–2. 10.4103/0366-6999.179797 27064047 PMC4831516

[13] Kreibich M Bavaria JE Branchetti E Brown CR Chen Z Khurshan F. Management of patients with coronary artery malperfusion secondary to type A aortic dissection. Ann Thorac Surg. (2019) 107:1174–80. 10.1016/j.athoracsur.2018.09.065 30444990

[14] Irimie V Atieh A Kucinoski G Jankulovski A Zacher M Urbanski PP. Long-term outcomes after valve-sparing anatomical aortic root reconstruction in acute dissection involving the root. J Thorac Cardiovasc Surg. (2020) 159:1176–84.e1. 10.1016/j.jtcvs.2019.04.036 31128903

[15] Mazzucotelli JP Deleuze PH Baufreton C Duval AM Hillion ML Loisance DY. Preservation of the aortic valve in acute aortic dissection: long-term echocardiographic assessment and clinical outcome. Ann Thorac Surg. (1993) 55:1513–7. 10.1016/0003-4975(93)91100-2 8512404

[16] Ro SK Kim JB Hwang SK Jung SH Choo SJ Chung CH. Aortic root conservative repair of acute type A aortic dissection involving the aortic root: Fate of the aortic root and aortic valve function. J Thorac Cardiovasc Surg. (2013) 146:1113–8. 10.1016/j.jtcvs.2012.08.055 22995725

