# Supplementary material for: Perioperative and Short-Term Outcomes of Sinus Replacement and Conservative Repair for Aortic Root in Acute Type A Aortic Dissection: A Prospective Cohort Study
Source: Front Cardiovasc Med. 2022 May 19;9:880411. doi: 10.3389/fcvm.2022.880411 (PMC9160325; doi:10.3389/fcvm.2022.880411)
Supplement: Supplementary file 1 [file Table_1.DOCX]

# Supplementary Table I Propensity Score Model AUC Analysis and Hosmer Lemeshow Test Result

| Index | Model AUC  Model AUC (95%CI) | Model AUC  (P Value) | Hosmer Lemeshow  Chi-Square Value | Hosmer Lemeshow  Test P Value |
| --- | --- | --- | --- | --- |
| Propensity Score Model | 0.67 (0.6164,0.7235) | <0.0001 | 8.4829 | 0.3878 |

# Adjusting：age, coronary heart disease, prior chronic renal insufficiency, open heart surgery, organ malperfusion syndrome, location of initial tear, type of coronary involvement, preoperative lactic acid and arch management

# 1.AUC P Value <0.05 is considered that the prediction is significant

# 2.Calibration (HL test) quantifies how close the predicted probabilities match the actual experience

# 3.HL test low p values represent lack of fit
