# Supplementary material for: Perioperative and Short-Term Outcomes of Sinus Replacement and Conservative Repair for Aortic Root in Acute Type A Aortic Dissection: A Prospective Cohort Study
Source: Front Cardiovasc Med. 2022 May 19;9:880411. doi: 10.3389/fcvm.2022.880411 (PMC9160325; doi:10.3389/fcvm.2022.880411)
Supplement: Supplementary file 2 [file Table_2.DOCX]

Supplementary Table II Absolute standardized mean differences between the two groups regarding baseline characteristics.

| **Variables** | **SMD**  **Before matching** | **SMD**  **After matching** |
| --- | --- | --- |
| Age, year | 0.112 | 0.003 |
| Male | 0.065 | 0.000 |
| BMI（Kg/m^2） | 0.026 | 0.030 |
| HT | 0.199 | 0.253 |
| DM | 0.048 | 0.153 |
| COPD | 0.065 | 0.069 |
| CAD | 0.172 | 0.063 |
| CRI | 0.093 | 0.045 |
| Previous heart surgery | 0.053 | 0.069 |
| VMS | 0.181 | NA |
| Initial tear - Arch | 0.224 | 0.018 |
| Initial tear - DTA | 0.132 | 0.045 |
| Type of CAI (left) - A | 0.225 | 0.065 |
| Type of CAI (right) -A | 0.303 | 0.084 |
| Type of CAI (right) -B | 0.112 | 0.067 |
| Type of CAI (right) -C | 0.125 | 0.098 |
| Scr, μmol/L | 0.056 | 0.044 |
| Lac,mmol/L | 0.002 | 0.011 |
| GPT, IU/L | 0.085 | 0.074 |
| TnI, ng/ml | 0.020 | 0.065 |
| Root diameter, mm | 0.225 | 0.051 |
| AR- Mild | 0.002 | 0.014 |
| AR- Moderate | 0.316 | 0.104 |
| AR- Moderate-severe | 0.131 | 0.083 |
| AR- severe | 0.175 | 0.091 |
| Arch repair -TAR | 0.017 | 0.000 |
| Arch repair -HAR | 0.011 | 0.037 |
| DTA management-FET | 0.212 | 0.108 |
| DTA management-Endovascular stent | 0.192 | 0.053 |
| SP-LCS | 0.447 | 0.449 |
| SP-RCS | 0.863 | 0.787 |
| SP-NCS | 1.792 | 1.869 |
| Commissure reattachment | 0.056 | 0.015 |
| CABG | 0.086 | 0.101 |
| CPB duration, min | 0.032 | 0.001 |
| Cross-clamp duration, min | 0.114 | 0.058 |
| HCA duration, min | 0.064 | 0.009 |
| Operation duration, hour | 0.130 | 0.091 |

SMD,Standardized mean difference; HT, hypertension; CAD, coronary artery disease; DM, diabetes mellitus; CRI, chronic renal insufficiency; VMS, visceral malperfusion syndrome; aAO, ascending aorta; DTA, descending thoracic aorta; CAI, coronary artery involvement; Scr, serum creatinine; Lac, lactic acid; IQR, Interquartile Range; GPT, glutamic-pyruvic transaminase; TnI, troponin I; AR, aortic regurgitation；SP, sinus plasty; LCS, left coronary sinus; RCS, right coronary sinus; NCS, none coronary sinus; HAR, hemi-arch replacement; TAR, total arch replacement; DTA, descending thoracic aorta; FET, frozen elephant trunk; CABG, coronary artery bypass grafting; CPB, cardiopulmonary bypass; HCA, hypothermic circulatory arrest.
