# Supplementary material for: Perioperative and Short-Term Outcomes of Sinus Replacement and Conservative Repair for Aortic Root in Acute Type A Aortic Dissection: A Prospective Cohort Study
Source: Front Cardiovasc Med. 2022 May 19;9:880411. doi: 10.3389/fcvm.2022.880411 (PMC9160325; doi:10.3389/fcvm.2022.880411)
Supplement: Supplementary file 3 [file Table_3.DOCX]

Supplementary Table III Multivariate subdistribution hazard model analysis of reoperation events (after controlling for death)

| Vaviables | Reference | P value | aHR | 95%CI |
| --- | --- | --- | --- | --- |
| Group | SP vs non-SP | 0.9977 | 0.000 | 0.000- NA |
| Age |  | 0.2851 | 1.088 | 0.932-1.271 |
| CAD | Yes vs No | 0.2080 | 11.255 | 0.260-487.429 |
| CRI | Yes vs No | 0.9997 | 0.000 | 0.000- NA |
| Previous heart surgery | Yes vs No | 0.9999 | 0.000 | 0.000- NA |
| VMS | Yes vs No | 1.0000 | 4.424 | 0.000- NA |
| Initial tear | Arch vs aAO | 0.1490 | 20.856 | 0.337-1290.905 |
| Initial tear | DTA vs aAO | 0.9996 | 0.000 | 0.000- NA |
| Type of CAI(left) | A vs no involvement | 0.9989 | 0.000 | 0.000- NA |
| Type of CAI (right) | A vs no involvement | 0.2410 | 8.839 | 0.231-337.630 |
| Type of CAI (right) | B vs no involvement | 0.9996 | 0.000 | 0.000- NA |
| Type of CAI (right) | C vs no involvement | 1.0000 | 2.020 | 0.000- NA |
| Lac |  | 0.9140 | 0.994 | 0.890-1.110 |
| Arch repair | TAR vs none | 1.0000 | 3.449 | 0.000- NA |
| Arch repair | HAR vs none | 0.9999 | 194.428 | 0.000- NA |

aHR, adjusted hazard ratio; CI: confident interval; SP, sinus plasty; CAD, coronary artery disease; CRI, chronic renal insufficiency; VMS, visceral malperfusion syndrome; aAO, ascending aorta; DTA, descending thoracic aorta; CAI, coronary artery involvement; Lac, lactic acid; TAR, total arch replacement; HAR, hemi-arch replacement.

* Competing risk model was constructed by subdistribution hazard function.
