# Supplementary material for: Perioperative and Short-Term Outcomes of Sinus Replacement and Conservative Repair for Aortic Root in Acute Type A Aortic Dissection: A Prospective Cohort Study
Source: Front Cardiovasc Med. 2022 May 19;9:880411. doi: 10.3389/fcvm.2022.880411 (PMC9160325; doi:10.3389/fcvm.2022.880411)
Supplement: Supplementary file 4 [file Table_4.docx]

Supplementary Table IV Operative characteristics and outcomes of three groups

| Variable | Sinus replacement  n=187 | Adventitial inversion  n=179 | Neomedia n=21 | P |
| --- | --- | --- | --- | --- |
| Arch repair (n, %) |  |  |  | 0.74 |
| None | 4(2.1) | 3(1.7) | 0(0) |  |
| HAR | 8(4.3) | 7(3.9) | 2(9.5) |  |
| TAR | 175(93.6) | 169(94.4) | 19(90.5) |  |
| DTA management (n, %) |  |  |  | 0.20 |
| None | 20(10.7) | 24(13.4) | 4(19.0) |  |
| FET | 157(84.0) | 137(76.5) | 14(66.7) |  |
| Endovascular stent | 10(5.3) | 18(10.1) | 3(14.3) |  |
| CABG (n, %) | 34(18.2) | 23 (12.8) | 7 (33.3) | 0.06 |
| CPB duration, min  (X ̅±SD) | 184.7 ±61.9 | 182.7±83.8 | 180.3±26.0 | 0.94 |
| Cross-clamp duration, min (X ̅±SD) | 114.7±43.6 | 108.7±41.9 | 120.1±21.1 | 0.26 |
| HCA duration, min  (X ̅±SD) | 14.3±8.8 | 13.5±9.9 | 14.8±7.9 | 0.69 |
| Operation duration, hour, (X ̅±SD) | 6.3±1.7 | 6.6±2.1 | 5.9±1.0 | 0.15 |
| MV duration, hour (median, IQR) | 21.0  (13.0-56.0) | 21.0  (13.0-42.0) | 18.0  (10.0-28) | 0.32 |
| ICU stay, day (X ̅±SD) | 5.5±5.1 | 5.4±4.4 | 4.6±3.2 | 0.68 |
| Operative mortality (n, %) | 6(3.2) | 12(6.7) | 0(0.0) | 0.11 |
| Restarted CPB for root bleeding (n, %) | 3(1.6) | 13(7.3) | 1(4.8) | 0.07 |
| PMI (n, %) | 0(0.0) | 1(0.6) | 0(0.0) | 0.46 |
| Reoperation for bleeding (n, %) | 2(1.1) | 5(2.8) | 0(0.0) | 0.32 |
| IABP (n, %) | 0(0.0) | 1(0.6) | 0(0.0) | 0.46 |
| ECMO (n, %) | 0(0.0) | 4(2.2) | 0(0.0) | 0.04 |
| Stroke (n, %) | 1(0.5) | 7(3.9) | 0(0.0) | 0.04 |
| CRRT (n, %) | 12(6.4) | 10(5.6) | 1(4.8) | 0.92 |
| Paraplegia (n, %) | 0(0.0) | 6(3.4) | 0(0.0) | 0.01 |

SR, sinus replacement; HAR, hemi-arch replacement; TAR, total arch replacement; DTA, descending thoracic aorta; FET, frozen elephant trunk; CABG, coronary artery bypass grafting; CPB, cardiopulmonary bypass; HCA, hypothermic circulatory arrest.

MV, mechanical ventilation; IQR, Interquartile Range; ICU, intensive care unit; PMI，myocardial infarction; IABP, intra-aortic balloon pump implantation; ECMO, extracorporeal membrane oxygenation; CRRT, continuous renal replacement therapy.
