# Supplementary material for: Perioperative and Short-Term Outcomes of Sinus Replacement and Conservative Repair for Aortic Root in Acute Type A Aortic Dissection: A Prospective Cohort Study
Source: Front Cardiovasc Med. 2022 May 19;9:880411. doi: 10.3389/fcvm.2022.880411 (PMC9160325; doi:10.3389/fcvm.2022.880411)
Supplement: Supplementary file 5 [file Data_Sheet_1.docx]

Supplementary text

Surgical technique of sinus plasty

The sinus plasty with preservation of native valve was performed as follow: ascending aorta was transected 1cm above sinotubular junction and the intimal flap of the involved sinus was removed maintaining a remanent edge of 5mm apart from the cusp insertion. Dissecting aortic root from surrounding tissue was troublesome so adventitia was reserved to omit separate an involved root. A patch deriving from artificial graft was trimmed to scallop shape similar to native valsalva sinus. 5-0 running polypropylene suture was used to sew the patch and remanent intima and adventitia together. Of note, the bottom of the patch should be sewed to aortic annulus (Figure 1 A, B and C). If we performed sinus plasty in left or right coronary sinus we would judge the severity of the dissected coronary artery by Neri classification. In type A, the intima of coronary orifice was trimmed into a button with a 5 mm circumferential cuff. The intimal button was attached snugly to the adventitia by running suture. After sinus plasty performed as mentioned above, a circular hole was created on the patch for receiving the coronary button. Then the button was re-implanted to the patch using a 5-0 running polypropylene suture (Figure 2 A, B and C). In type B and C, we selected coronary artery bypasses grafting (CABG) for security. The avulsed commissure was attached to adventitia using interrupted mattress suture with pledge. Then the root stump was prepared for proximal anastomosis.
