# Supplementary figures and images for: Perioperative and Short-Term Outcomes of Sinus Replacement and Conservative Repair for Aortic Root in Acute Type A Aortic Dissection: A Prospective Cohort Study
Source: Front Cardiovasc Med. 2022 May 19;9:880411. doi: 10.3389/fcvm.2022.880411 (PMC9160325; doi:10.3389/fcvm.2022.880411)

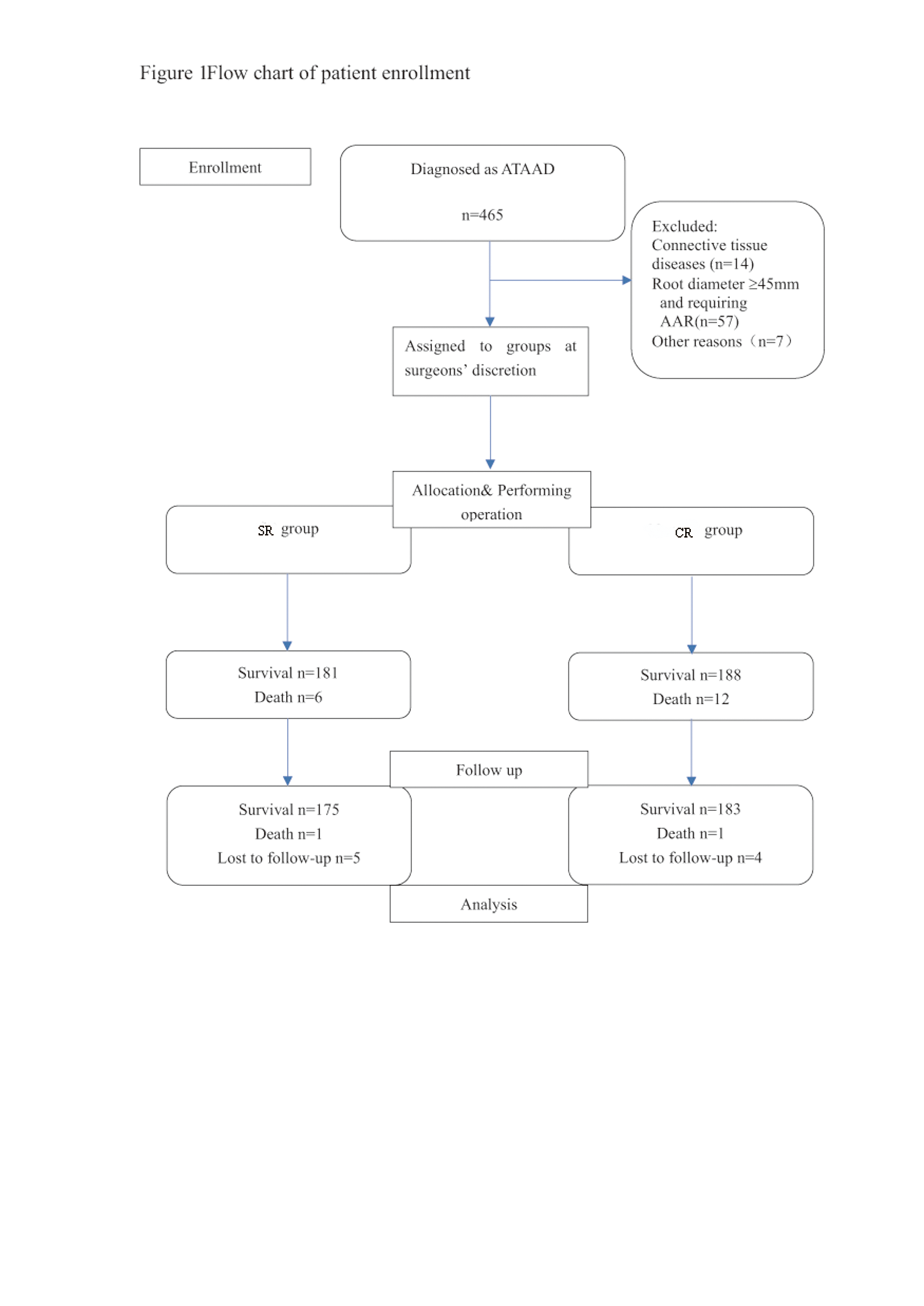

Supplement: Supplementary file 6 [file Image_1.TIF]
